# Higher Levels of Aflatoxin M1 Contamination and Poorer Composition of Milk Supplied by Informal Milk Marketing Chains in Pakistan

**DOI:** 10.3390/toxins8120347

**Published:** 2016-12-05

**Authors:** Naveed Aslam, Muhammad Yasin Tipu, Muhammad Ishaq, Ann Cowling, David McGill, Hassan Mahmood Warriach, Peter Wynn

**Affiliations:** 1 Graham Centre for Agricultural Innovation, Charles Sturt University, Wagga 2650, NSW, Australia; acowling@csu.edu.au (A.C.); pwynn@csu.edu.au (P.W.); 2 Quality Operation Laboratory, University of Veterinary and Animal Sciences, Lahore 54000, Punjab, Pakistan; yasintipu@uvas.edu.pk; 3 Agriculture Sector Linkages Program Dairy Project, University of Veterinary and Animal Sciences, Lahore 54000, Punjab, Pakistan; drishaq_rai@yahoo.com (M.I.); hassanwarriach71@yahoo.com (H.M.W.); 4 Faculty of Veterinary and Agricultural, University of Melbourne, Werribee 3030, VIC, Australia; david.mcgill@unimelb.edu.au

**Keywords:** AFM1, milk value chains, smallholder dairy production, milk composition, adulteration

## Abstract

The present study was conducted to observe the seasonal variation in aflatoxin M1 and nutritional quality of milk along informal marketing chains. Milk samples (485) were collected from three different chains over a period of one year. The average concentrations of aflatoxin M1 during the autumn and monsoon seasons (2.60 and 2.59 ppb) were found to be significantly higher (standard error of the difference, SED = 0.21: *p* = 0.003) than in the summer (1.93 ppb). The percentage of added water in milk was significantly lower (SED = 1.54: *p* < 0.001) in summer (18.59%) than in the monsoon season (26.39%). There was a significantly different (SED = 2.38: *p <* 0.001) mean percentage of water added by farmers (6.23%), small collectors (14.97%), large collectors (27.96%) and retailers (34.52%). This was reflected in changes in milk quality along the marketing chain. There was no difference (*p* = 0.178) in concentration of aflatoxin M1 in milk collected from the farmers (2.12 ppb), small collectors (2.23 ppb), large collectors (2.36 ppb) and retailers (2.58 ppb). The high levels of contamination found in this study, which exceed the standards set by European Union (0.05 ppb) and USFDA (0.5 ppb), demand radical intervention by regulatory authorities and mass awareness of the consequences for consumer health and safety.

## 1. Introduction

A significant proportion of food and food products worldwide is affected by mycotoxins (fungal secondary metabolites) each year [1]. Among these mycotoxins, aflatoxins (AFs) are the most toxic compounds. Aflatoxins (B1, B2, G1 and G2) are naturally occurring toxic substances with carcinogenic properties [2], and are produced mainly by *Aspergillus flavus*, *A. parasiticus* and *A. nominus* [3]. Aflatoxin B1 (AFB1) is the most important toxin in this group, acting as a mutagen and a carcinogen [4]. Aflatoxin M1 (AFM1), produced as a result of hydroxylation of AFB1 [3], is detected in the milk of animals and humans after the ingestion of feed contaminated with AFB1 [5]. AFB1 is dependent on the cytochrome P450 group of enzymes for its conversion to 8, 9 AFB1 epoxide, resulting in its carcinogenicity and acute cytotoxicity [6]. AFM1 also has toxic effects without the need for metabolic activation [3].

High-level exposure of humans to AFs for a short time may lead to fatal aflatoxicosis [7]. In one incident, several hundred people in Kenya fell ill, with 125 dying of acute aflatoxicosis after the consumption of highly contaminated food [8]. Liver cancer or hepatocellular carcinoma (HCC) is the main resulting disease [9]. AFs are also reported to be associated with reduced growth in children and disorders of the immune system [10]. Several hundred deaths of calves, chinchilla and buffaloes in Australia, Argentina and Pakistan respectively, provide examples of acute aflatoxicosis as a potential threat to animals [11,12,13]. Chronic exposure may lead to impaired production and reproduction and increased mortality rates in animals. Lactating animals and humans consuming AFs secrete AFM1 in their milk. This provides a potential health risk to their suckling offspring [10].

The European Union (EU-25) and South Asia collectively produce 44% of the global milk supply [14]. Between the years 2002 to 2007, world milk production increased by 13%. This was mainly due to greater contributions from Pakistan, China and India [14]. Furthermore, 150 million farm households worldwide engage in dairy production, mainly in developing countries where smallholder systems are dominant. This not only provides a source of food security for the poor and malnourished producers but also offers employment opportunities along milk value chains [14,15]. Around 97% of the total milk production in Pakistan is handled by informal chains, where smallholder dairy farmers, small, medium and large milk collectors and retailers market milk in its raw form [16]. Milk quality assurance is one of the major challenges faced by these informal chains which transport more than 30% of the country’s total produced milk from smallholder dairy producers to large retailers in cities [17]. Addition of water, chemicals and drug residues both intentionally and unintentionally has also been reported [16,17]. Recent surveys [18,19,20] have highlighted the presence of AFM1 in milk and milk products. However, informal milk marketing chains specifically have not been the focus of previous investigations of mycotoxin contamination in Pakistan.

The present study was conducted with the objectives of observing the concentration of AFM1 and changes in overall milk quality along the traditional marketing chains as milk passes from smallholder dairy farmers to the consumers in Pakistan. We also report on seasonal changes in AFM1 concentration and milk quality within these value chains.

## 2. Results

### 2.1. Milk Quality along the Milk Value Chains

**Figure 1:**
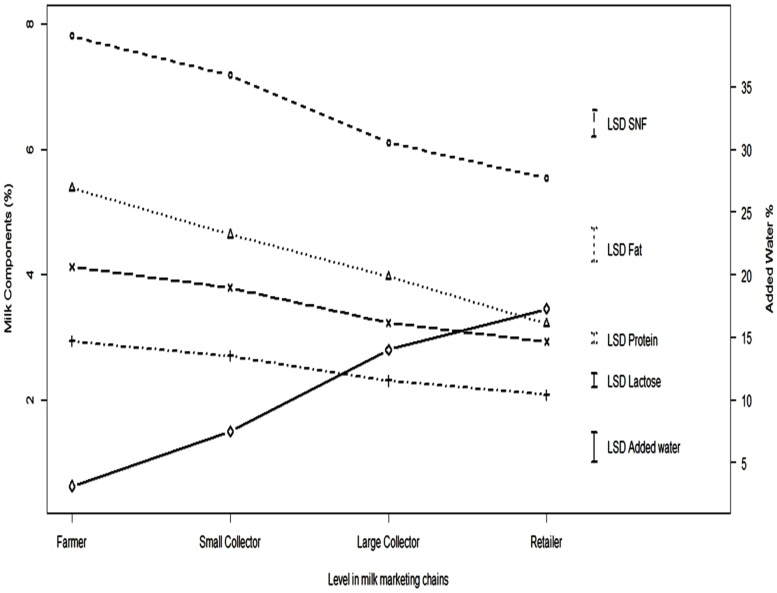
Variation in milk composition and total addition of water at different levels in informal milk marketing chains averaged across seasons for the year (data presented as means with least significant differences for each parameter). Note: ο, +, ∆, x and ◊ describe the values of solids not fat (SNF) lactose fat protein and added water respectively.

A continuous decline in milk quality with respect to solids not fat (SNF), fat, protein and lactose content, and an increase in AFM1, was observed as milk passed along the milk supply chains from farmers to retailers. Mean percentages of constituents in milk collected from farmers, small collectors, large collectors and retailers are presented in Figure 1. The differences in percentages of the normal milk constituents fat (standard error of the difference: SED = 0.27), protein (SED = 0.08), SNF (SED = 0.21) and lactose (SED = 0.11) along the milk marketing chains were highly significant (*p <* 0.001). The highest mean values for fat (5.37%), protein (2.93%), SNF (7.81%) and lactose (4.12%) were found in samples collected directly from the farmer. On the other hand, milk collected from retailers was observed to have the lowest average concentrations of milk constituents. The values of fat, protein, SNF and lactose in milk samples collected from retailers were 3.21%, 2.08%, 5.54% and 2.92% respectively. The highest mean percentage for added water was found with large collectors (12.99%). On the other hand, mean values for added water for farmers, small collectors and retailers were 6.23%, 8.74% and 6.56%, respectively.

There was a highly significant (SED = 2.38: *p <* 0.001) difference in the percentage of water added along steps in the milk chains. Water was added at every level in the form of ice or water to limit bacterial growth or to increase milk volume, respectively. A trend of increasing volumes of water being added (Figure 1) was observed as milk flowed down the chain. There was no significant difference for concentration of AFM1 observed between different points along the marketing chains, although total average concentration increased at each step. Mean values for concentrations of AFM1 in milk samples collected from farmers, small collectors, large collectors and retailers were 2.12, 2.22, 2.36 and 2.58 parts per billion (ppb, SED = 0.31; *p* = 0.178) respectively. The total number of positive and negative samples for AFM1, and samples exceeding US (0.5 ppb) standards, are given in Table 1.

### 2.2. Effect of Season on Aflatoxin M1 and Milk Composition

**Figure 2:**
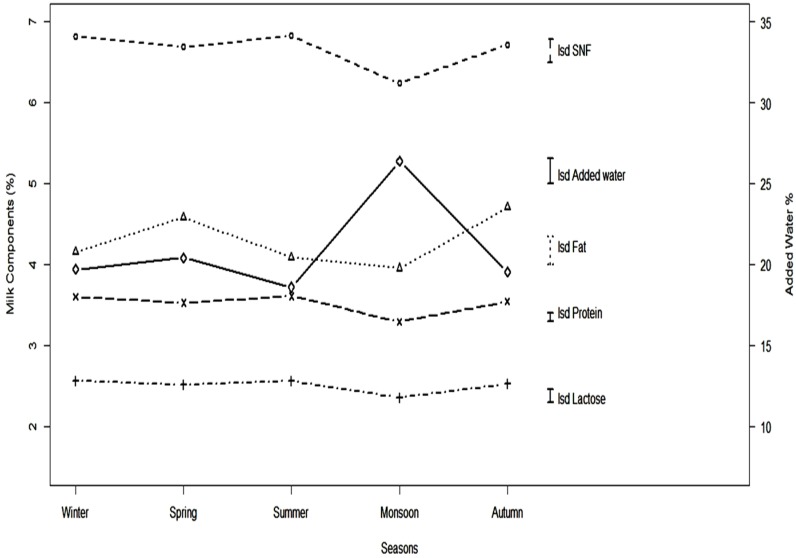
Variation in milk constituents and contaminants with season in informal milk supply chains. Note: ο, +, ∆, x and ◊ describe the values of SNF, lactose, fat, protein and added water respectively.

The average concentrations of AFM1 were 2.25, 2.04, 1.93, 2.59 and 2.60 ppb for winter, spring, summer, monsoon and autumn respectively (*p* = 0.003). The highest average concentrations of AFM1 were found in the autumn and monsoon seasons while the lowest average concentration was found in summer. Seasonal differences in fat (SED = 0.17), protein (SED = 0.05), lactose (SED = 0.08) and SNF (SED = 0.14) were highly significant (*p <* 0.001). Figure 2 shows the pattern of variation in milk quality, water addition and concentration of AFM1 in different seasons. The highest mean values for protein (2.56%), SNF (6.83%) and lactose (3.61%) content were observed in summer whereas the lowest average values (2.35%, 6.24% and 3.29% respectively) were observed in the monsoon season. On the other hand, the highest average value for fat (4.71%) was observed in autumn and the lowest (3.95%) in the monsoon season.

Differences between the values for mean added water (%) in various seasons of the year were highly significant (SED = 1.54: *p <* 0.001). The highest percentage (26.39) was found in the monsoon season whereas the lowest value for addition of water (18.59) was observed in summer. This was the reason for the lower milk constituent concentrations in the monsoon season and higher levels in summer.

The total number of samples exceeding US standards (0.5 ppb) according to the season are detailed in Table 2.

### 2.3. District-Wise Changes in Milk Quality

The AFM1 concentration in milk was significantly (SED = 0.23: *p <* 0.001) different across the districts studied. The average concentration of AFM1 for one complete year across the marketing chains in the district of Okara (1.72 ppb) was lower than the concentrations in the districts of Kasur (2.61 ppb) and Pakpattan (2.53 ppb). There was a highly significant (*p <* 0.001) difference in the concentrations of protein, SNF, lactose in the districts of Kasur, Okara and Pakpattan. The percentage of fat did not differ (*p* = 0.064) between districts. On the other hand, the percentage of water addition in milk was significantly different (*p <* 0.001). Table 3 shows the mean values for percentages of fat, protein, SNF, lactose and added water in all three districts.

No sample was found to be contaminated with any adulterants (hypochlorite, soap, sorbitol, cane sugar, sodium chloride, carbonates, urea, formaldehyde, boric acid, starch, H_2_O_2_ and QAC) except water.

## 3. Discussion

Identification of levels of AFM1 along the milk chain that exceed internationally accepted levels is a major concern for the consumers of milk in Pakistan. The tendency for these concentrations to increase along the chain may be the result of more highly contaminated milk being added by other chain participants (farmers, small and large collectors, and retailers) and/or the addition of AF-contaminated water at every level. AFs are slightly soluble (10–30 µg∙mL^−1^) in water [21] and are highly stable in that medium [22]. Natural occurrence of AFs has also been reported in stored water by Paterson, et al. [23]. Milk samples analyzed in the present study were mixed samples collected both from cows and buffaloes. One representative sample was collected each time after mixing the milk harvested from all of the animals before collection.

The contamination levels of AFM1 observed in our survey were higher than those observed by Sadia et al. [18] as detailed in Table 6. They reported concentrations of AFM1 as 0.18, 0.47 and 0.11 ppb in milk samples collected from local shops, households and dairy farms respectively across Punjab province. A number of surveys [2,19,24,25] have been conducted (see Table 6 below for detailed results) in the past for the prevalence of AFM1 in milk in Pakistan but none of them have focused specifically on informal milk marketing chains supplying milk to the big cities.

The pattern of fat percentage was different from other constituents, showing its highest mean concentrations in autumn instead of summer. This may be due to the missing data for fat analyses in summer. Incomplete homogenization of the milk samples caused separation of fat content with some samples. Thus fat could not be analyzed in these samples using Milkoscan. The concentration of AFM1 in milk was lower in a survey of milk marketing chains conducted in Punjab by Hussain and Anwar [24]. The highest mean concentration in their study was found to be in the month of January, while the lowest contamination was observed in August (Table 6). This contrasts with the higher mean levels found in the monsoon and autumn (2.59 and 2.60 ppb), with the lowest mean concentration identified in summer (1.93 ppb) in the present study. The difference between the two studies may be because of the different sources of sample collection. The concentrations found in our survey were 3.87–5.21 times higher than limits set by the United States (0.5 ppb) and 38.7–52.1 times higher than the European Union (EU) standard (0.05 ppb) whereas the highest concentration found by [24] was 10 times the EU standards and exactly the same as US standards. Season/weather is one of the most important factors contributing to the contamination of milk with AFs, as is the year of the study. In our study, samples were collected from October 2012 to September 2013 and this is the most recent survey of its kind published so far.

Concentrations of AFM1 in milk samples in the present study are higher than those reported by Iqbal et al. [20]. The contamination of raw milk samples was observed to be higher in winter than in summer in their study (Table 6). Again, this may be due to differences in the source of samples. Samples in the present study were collected from the districts of Lahore, Kasur, Okara and Pakpattan. On the other hand, samples were collected from the districts of Sheikhupura, Faisalabad, Sahiwal, Jhang, Gujranwala, Sargodha and Chakwal in the study by Iqbal and Asi [19]. Although the districts studied in each report are located close to each other, their report did not focus specifically on informal milk marketing chains, but rather on collected samples mainly from small and large dairies and dairy farmhouses and milk collection sites. In addition, the proportion of samples collected from each site was not mentioned. The higher contamination during the monsoon season observed in the current study clearly indicated the effect of high temperature and humidity on the accumulation of fungal contamination and mycotoxins in the feed provided for animals which were then transferred to the milk. A comprehensive summary of studies conducted to report on AFM1 contamination in milk in Pakistan is shown in Table 6 below.

Lateef, et al. [26] evaluated milk samples from canteens of hospitals in the large city of Faisalabad (Punjab). Percentages of fat, protein, SNF and total solids were 1.52, 1.20, 4.98 and 6.54 respectively. Adulteration with water, urea, formalin, hydrogen peroxide and cane sugar was observed in 93.3%, 86.6%, 46.6%, 13.3% and 93.3% of the samples respectively. In another study [27], milk composition and adulteration was observed in cafeterias of various educational institutes and public places in the same city (Faisalabad). Furthermore, values for protein (1.12% and 1.33%), fat (2.6% and 1.40%), SNF (5.10% and 4.77%) and total solids (7.18% and 6.17%) in educational institutes and public places respectively, were less than half (46%–48%) for protein and fat respectively and about 75% of the value for SNF observed in the present survey. In the same study adulteration with urea, formalin and cane sugar in educational institutes and public places was found in 63% and 87%, 23% and 27%, 87% and 97% of the samples respectively. Three percent of the samples collected from public places were adulterated with hydrogen peroxide. Clearly adulteration of milk is widespread and more serious in many parts of Pakistan than observed in the current study.

Unlike previous studies [26,27], no adulterant was found in the present study except water (ice), which is an essential part of informal milk marketing chains added in the form of ice to maintain lower temperature of milk during transportation. Milk obtained from the informal milk marketing chains of the present study contained higher levels of solids and no intentional chemical adulterants as reported by Faraz et al. [27] and Lateef et al. [26]. This shows clearly that milk reaching the consumer can vary significantly in quality across districts and urban centers in Pakistan. This may reflect differences in the rigor of the regulatory framework in place to control milk composition or might simply reflect a more direct route for milk transferred from the farmer to consumer in some districts. Irrespective of site of sampling, it is hard to trace the origin of milk used at most of the cafeterias in big cities.

As far as milk composition and levels of adulteration are concerned, the informal milk marketing chains studied here are supplying milk of acceptable quality to large cities across the year. On the other hand, contamination of milk with high levels of AFM1 poses a serious health issue for consumers. This issue can be resolved by sourcing concentrate feed devoid of mycotoxins and by promoting awareness, education and training of the personnel involved in milk production and distribution and most importantly, the consumer.

One of the aspects that could not be pursued in our survey was analysis of water/ice used to dilute milk. This was because the adulteration was generally covert, in the interests of maintaining confidentiality. There is still a need to evaluate milk sampled along these informal milk supplying chains for other mycotoxins residues such as ochratoxin, zearalenone, deoxynivalenol and fumonisins. These studies should extend to peri-urban production systems as they are the other major purveyors of milk to the urban population.

## 4. Conclusions

The present study has highlighted the importance of the regulation of the quality of milk produced by smallholder dairy producers for the consumers in Pakistan. The key outcome of the study was the finding that milk samples obtained from operators in milk supply chains collecting from small-holder farmers across seasons in three regions of Punjab province Pakistan contain significant concentrations of AFM1. These levels exceed maximum accepted levels in the EU and USFDA by 5- to 50-fold. These milk samples were also adulterated with water resulting in a dilution of the key nutritional components protein, fat and solids by approximately 50%. This situation could be improved by creating public awareness of the widespread nature of mycotoxin contamination and milk adulteration with poor quality water. This then needs to lead to the education of those directly involved in the production, collection/distribution and selling of milk as well as legislators controlling the industry about the health risks associated with these practices.

## 5. Materials and Methods

### 5.1. Experimental Site

Three informal milk marketing chains from rural Kasur, Okara and Pakpattan districts of province Punjab were identified for this survey. All three chains started from smallholder dairy farmers in villages and ended at specialized urban milk retail shops in metropolitan Lahore. Milk was handled by a series of small and large collectors/distributors.

### 5.2. Traditional Milk Marketing Chains

**Figure 3:**
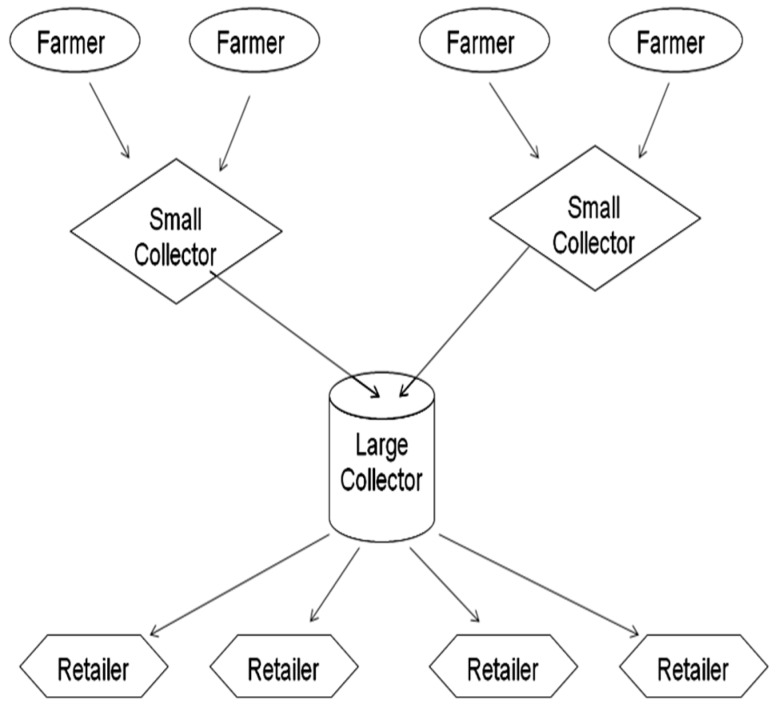
A typical structure for an informal milk marketing chain in Pakistan.

Informal milk marketing chains in Pakistan are very complicated, but they do have some common features. In these chains fresh, unpackaged milk passes through many hands (farmers, small collectors, large collectors, retailers, bakers and confectioners), using only the most rudimentary cool chain system before reaching the consumer. A typical informal milk marketing chain of this type is described in Figure 3 [33,34].

### 5.3. Sample Collection

Milk samples (100 mL) were collected monthly from October 2012 to September 2013. A total of six to eight farmers, three small collectors, one large collector and four retailers were selected in each chain for sample collection. A total of 485 milk samples from the three informal milk marketing chains were collected from the bulk tank milk of farmers (214), small collectors (98), large collectors (35) and retailers (138). One complete year (12 months) was divided into five seasons: winter, spring, summer, monsoon and autumn to determine the seasonal changes in milk quality. The winter season was classified as the months from December to February; spring from March to April; summer from May to July; monsoon from August to September and autumn from October to November.

### 5.4. Experimental Parameters

#### 5.4.1. Milk Composition

Milk composition analysis was conducted for milk fat, solids not fat (SNF), protein, lactose and added water with a Lactoscan-S Milk Analyzer (50 W, Milkotronic Ltd., Nova Zagora, Bulgaria) in the World Trade Organization (WTO) Quality Operation Laboratory at University of Veterinary and Animal Sciences (UVAS) Lahore. This analyzer calculated the added water by measuring differences in freezing point of the samples. The freezing point of normal bovine milk is −0.525 °C and so any sample freezing above this temperature was designated as having water added. Water is added to milk in the form of ice to preserve its quality and sometimes added directly to increase the volume of the product.

#### 5.4.2. Milk Adulterants

Milk samples were analyzed for hypochlorite, soap, sorbitol, cane sugar, sodium chloride, carbonates, urea, formaldehyde, boric acid, starch, hydrogen peroxide (H_2_O_2_) and quaternary ammonium compounds (QAC) using a Milk Adulterant Testing (MAT) kit designed by the WTO Quality Operation Laboratory UVAS, Lahore, Pakistan [35]. The sensitivity for the MAT is 0.05% for all adulterants except for formalin which had a sensitivity of 1:40,000.

#### 5.4.3. Aflatoxin M1 Analysis

AFM1 was measured by direct competitive enzyme-linked immunosorbent assay (ELISA) using the AgraQuant^®^ AflatoxinM1 Fast ELISA kits supplied by Romer Labs^®^ Singapore Pty Ltd., Singapore, according to the assay method provided with the kits. The limit of detection of AFM1 in fresh milk was 89 parts per trillion. The recovery of AFM1 of the assay was 83%–99%. Three analysts using two different batches of test kits and cross-reactivities with aflatoxin B1, B2, G1 and G2 found values of 88%, 27%, 11.5% and 4.7% respectively according to the information provided by the manufacturer.

##### Sample Preparation/Extraction

A 5 mL milk sample was incubated for 30 min at 4 °C. The sample was then centrifuged at 3000× *g* at same temperature for 10 min. The milk serum below the fat layer was diluted 20 times with double distilled water. Following this, 0.4 mL of the diluted milk serum was mixed with 0.1 mL of 100% methanol (4:1) and used in the ELISA.

##### ELISA Assay Procedure

One aflatoxin M1-specific antibody coated well was used for each standard (0, 100, 200, 500, 1000, 2000 ngL^−1^) or sample. To each dilution well, 200 µL of the aflatoxin M1-specific monoclonal antibody-enzyme conjugate was dispensed. Then, 100 µL of each standard or sample was placed into the appropriate dilution well. Each well was then mixed carefully. These solutions (100 µL) were then dispensed into the corresponding antibody coated microwell. Samples were incubated at room temperature (18–30 °C) for 20 min.

Microwell strips were then placed into an automatic ELISA washer (ELx50, BioTek, Winooski, VT, USA), washed 5 times and then drained using absorbent towels to dry residual solution. Enzyme substrate (100 µL) was dispensed into each well and incubated for 10 min in the dark. Stop solution (100 µL) was dispensed into each well. At this time, the color changed from blue to yellow. Optical densities (OD) were recorded in a microwell plate reader (Multiskan^®^ EX, Thermo Scientific, Waltham, MA, USA) at a wavelength of 450 nm.

### 5.5. Statistical Analyses

Monthly data were collectively analyzed for each season to identify seasonal changes in milk composition and contamination status. AFM1, added water, fat, lactose, protein, and SNF were analyzed statistically using linear mixed models with all two-way interactions between three districts (Kasur, Okara and Pakpattan) with type (farmer, small collector, large collector and retailer) and season as fixed effects, and district.source as a random effect. Source refers to the specific farmer, small collector, large collector and retailer within each type. The model with district.source as a random effect had a smaller Akaike’s information criterion (AIC) than models with random effects involving nesting, that in some ways better reflected the nesting of the study design. Sequential backward elimination was used, due to the unbalanced design, to reduce the model until only statistically significant terms (and their lower order components) remained. Residual plots were examined to ensure that the assumptions of normality and homogeneity of variance were met.

The number of samples that could not be analyzed for AFM1, added water and fat were 1, 54 and 83 respectively. On the other hand, 51 values were missing for protein, lactose and SNF each. The time course of missing data affected some interaction analyses: for example, it was not possible to estimate the three-way interaction for lactose, protein and SNF. The all two-way interactions model was the most comprehensive model which could be fitted to all response variables. Data are cited as means together with their Fisher’s least significant differences (LSDs) in Figure 1 and Figure 2 to compare individual treatment means. Standard errors of differences (SEDs) are stated elsewhere. Statistical significance was attained if the probability value (*P*) was less than 5%. Genstat 16th edition (Hemel Hempstead, UK) was used for all analyses [36].

## Figures and Tables

**Table 1:** Number of samples exceeding US standards for aflatoxin M1 (AFM1) at various levels of informal milk marketing chains in Pakistan.

Level in Milk Marketing Chains	Negative Samples	Positive Samples	Percentage of Samples Exceeding US Standards (0.5 Parts per Billion, ppb)
Farmers	14	200	78.50
Small collectors	3	95	89.80
Large collectors	0	35	100
Retailers	0	138	95.65
Total	17	468	87.22

**Table 2:** Total number of positive and negative samples for AFM1 concentration and samples exceeding US standards by season.

Season	Negative Samples	Positive Samples	Percentage of Samples Exceeding US Standards (0.5 ppb)
Winter	4	121	87.20
Spring	1	86	88.50
Summer	3	120	88.62
Monsoon	1	77	87.18
Autumn	8	64	83.33
Total	17	468	87.22

**Table 3:** Mean percentages of fat, protein, SNF, added water and lactose in milk of informal supply chains originating from different districts.

Districts	Fat %	Protein %	SNF %	Added Water %	Lactose %
Okara	4.527	2.570	6.818	19.65	3.598
Pakpattan	4.175	2.568	6.850	18.51	3.618
Kasur	4.231	2.367	6.303	24.61	3.327
SED	0.18	0.07	0.10	2.12	0.19
*p*-Values	0.064	<0.001	<0.001	<0.001	<0.001

Highly significant interactions between districts and seasons for added water, protein, lactose and SNF have been observed and detailed in Table 4 below. SED: standard error of the difference.

**Table 4:** Mean values for added water, protein, SNF and lactose in different seasons across all three districts.

Milk Component	Season District	Winter	Spring	Summer	Monsoon	Autumn	SED	*p* Value
Added Water %	Okara	17.54	20.19	17.39	22.59	20.52	2.756	0.037
	Pakpattan	18.26	16.16	15.82	27.21	15.10		
	Kasur	23.27	24.91	22.56	29.37	22.93		
Lactose %	Okara	3.734	3.516	3.753	3.503	3.485	0.1348	0.019
	Pakpattan	3.655	3.721	3.716	3.254	3.745		
	Kasur	3.412	3.350	3.355	3.124	3.392		
Protein %	Okara	2.659	2.519	2.667	2.509	2.494	0.0940	0.015
	Pakpattan	2.593	2.642	2.638	2.313	2.655		
	Kasur	2.428	2.377	2.376	2.232	2.422		
SNF %	Okara	7.072	6.673	7.100	6.631	6.612	0.2528	0.018
	Pakpattan	6.919	7.045	7.037	6.157	7.094		
	Kasur	6.468	6.344	6.347	5.925	6.434		

Mean concentrations (ppb) for AFM1 at different levels of informal milk marketing chains, in various seasons of the year and in three different districts (i.e., Okara, Kasur and Pakpattan) have been given below in Table 5.

**Table 5:** Mean concentrations (ppb) of AFM1 at different levels of milk marketing chains, in various seasons and in different districts.

AFM1 in seasons of the year (ppb)	**Winter**	**Spring**	**Summer**	**Monsoon**	**Autumn**
	2.25	2.04	1.93	2.59	2.60
AFM1 at various level of marketing chains (ppb)	**Farmers**	**Small Collectors**	**Large Collector**	**Retailers**	
	2.12	2.22	2.36	2.58	
AFM1 in different districts (ppb)	**Kasur**	**Okara**	**Pakpattan**		
	2.61	1.72	2.53		

**Table 6:** Summary of research conducted estimating AFM1 contamination in milk in Pakistan with respect to species and season (in some studies: winter denoted by W and summer by Su).

Species	Minimum Concentration ppb	Maximum Concentration ppb	Mean Concentration ppb	No. of Samples Analyzed	References
Mixed (cattle and buffalo)	0.01	0.7	0.371	168	[24]
Buffalo	0.050 (W)	0.200 (W)	0.091 (W)	97	[2]
	0.025 (Su)	0.105 (Su)	0.042 (Su)		
Cattle	0.065–0.150 (W)	0.150 (W)	0.089 (W)	76	[2]
	0.014 (Su)	0.095 (Su)	0.022 (Su)		
Goat	0.008 (W)	0.090 (W)	0.069 (W)	62	[2]
	0.009 (Su)	0.088 (Su)	0.018 (Su)		
Sheep	0.010 (W)	0.088 (W)	0.079 (W)	75	[2]
	0.012 (Su)	0.069 (Su)	0.024 (Su)		
Camel	0.012 (W)	0.064 (W)	0.058 (W)	46	[2]
	0.005 (Su)	0.081 (Su)	0.010 (Su)		
Mixed (cattle and buffalo)	0.00	0.845	0.151	107	[19]
Mixed (cattle and buffalo)	0.00	0.89	0.049	104	[20]
UHT Milk	0.00	0.51	0.07	84	[20]
Buffalo	NA	NA	0.027	360	[25]
Cow	NA	NA	0.044	120	[25]
Mixed (cattle and buffalo)	0.00	0.040	0.018	21	[28]
Cow	0.00	0.084	0.037	84	[29]
Buffalo	0.00	0.350	0.043	94	[29]
Mixed (cow and buffalo)	0.69	100.04	17.38	84	[30]
Mixed (cow and buffalo)	0.00	1.9	0.252	232	[18]
Mixed (cow and buffalo)	0.00	7.28	2.23	485	[31]

Source: Aslam and Wynn [32].
